# 人源化CD19 CAR-T细胞治疗儿童及青少年复发难治急性淋巴细胞白血病患者的疗效及安全性

**DOI:** 10.3760/cma.j.issn.0253-2727.2022.07.005

**Published:** 2022-07

**Authors:** 诗媛 王, 李娜 赵, 海 程, 明 施, 伟 陈, 昆明 齐, 财 孙, 雪 王, 江 曹, 开林 徐

**Affiliations:** 徐州医科大学附属医院血液科，徐州 221002 Department of Hematology, The Affiliated Hospital of Xuzhou Medical University, Xuzhou 221002, China

**Keywords:** 嵌合抗原受体, 人源化, CD19, 难治复发, 急性淋巴细胞白血病, Chimeric Antigen Receptor, Humanized, CD19, Relapsed/refractory, Acute lymphoblastic leukemia

## Abstract

**目的:**

探讨人源化CD19 CAR-T细胞（hCART19s）治疗儿童及青少年复发、难治性急性淋巴细胞白血病（R/R ALL）的疗效及安全性，研究影响其预后的相关因素。

**方法:**

筛选2016年5月至2021年9月徐州医科大学附属医院入组NCT02782351临床试验中31例25岁以下儿童及青少年R/R ALL患者的临床资料，评价hCART19s在年轻患者中的疗效及安全性。

**结果:**

hCART19s输注后1个月，短期疗效评估显示27例（87.1％）患者获得完全缓解（CR）或完全缓解兼部分血细胞计数缓解（CRi）。治疗期间，20例（64.5％）患者出现1～2级细胞因子释放综合征（CRS），4例（12.9％）出现3～4级CRS；2例患者出现1级神经系统毒性。中位随访19.3（2.2～62.4）个月，中位无事件生存（EFS）率和总生存（OS）率分别为15.7（95％ *CI* 8.7～22.5）个月和32.2（95％ *CI* 10.6～53.9）个月。桥接移植患者EFS和OS率均高于未桥接移植患者［EFS：（75.0±12.5）％对（21.1±9.4）％，*P*＝0.010；OS：（75.0±12.5）％对（24.6±10.2）％，*P*＝0.012］；既往治疗线数>3次的患者EFS和OS率明显低于治疗线数≤3次的患者［EFS：0对（49.5±10.4）％，*P*<0.001；OS：0对（52.0±10.8）％，*P*<0.001］。截止随访终点，13例患者出现CD19阳性（CD19^+^）复发，1例出现CD19阴性（CD19^−^）复发。

**结论:**

hCART19s可有效治疗儿童及青少年R/R ALL患者，其严重不良事件发生率较低。桥接移植、治疗线数可对患者长期疗效及预后产生影响。

## Abstract (en)

**Objective:**

To investigate the efficacy and safety of humanized CD19-specific chimeric antigen receptor T cells（hCART19s）in treating children and young adults with relapsed/refractory acute lymphoblastic leukemia（R/R ALL）and to analyze relevant factors affecting its curative effect and prognosis.

**Methods:**

We conducted a single-center clinical trial involving 31 children and young adult patients with R/R B-ALL who were treated with humanized CD19-specific CAR-T cells（hCART19s）from May 2016 to September 2021.

**Results:**

Results showed that 27（87.1％）patients achieved complete remission（CR）or CR with incomplete count recovery（CRi）one month after CAR-T cell infusion. During treatment, 20（64.5％）patients developed grade 1–2 cytokine release syndrome（CRS）, and 4（12.9％）developed grade 3 CRS. Additionally, two patients had grade 1 neurological events. During the follow-up with a median time of 19.3 months, the median event-free survival（EFS）was 15.7 months（95％ *CI* 8.7–22.5）, and the median overall survival（OS）was 32.2 months（95％ *CI* 10.6–53.9）. EFS and OS rates were higher in patients who have undergone hemopoietic stem cell transplantation（HSCT）than in those without［EFS:（75.0 ± 12.5）％ *vs*（21.1 ± 9.4）％, *P*＝0.012; OS:（75.0 ± 12.5）％ *vs*（24.6 ± 10.2）％, *P*＝0.035］. The EFS and OS rates were significantly lower in patients with >3 treatment lines than in those with <3 treatment lines［EFS: 0 *vs*（49.5±10.4）％, *P*<0.001; OS: 0 *vs*（52.0±10.8）％, *P*<0.001］. To the cutoff date, 12 patients presented with CD19^+^ relapse, and 1 had CD19^−^ relapse.

**Conclusion:**

hCART19s are effective in treating pediatric and young adult R/R ALL patients, with a low incidence of severe adverse events and reversible symptoms. Following HSCT, the number of treatment lines can affect the long-term efficacy and prognosis of pediatric and young adult R/R ALL patients.

随着诊疗技术的改进，儿童及青少年急性淋巴细胞白血病（ALL）患者的缓解率和长期生存率明显提高，但仍有部分复发/难治（R/R）患者难以获益[1]–[3]。2017年，全球首个靶向CD19的嵌合抗原受体T（CAR-T）细胞产品tisagenlecleucel获批上市，用于治疗儿童和青少年R/R ALL[4]。目前已有多款靶向CD19的CAR-T细胞产品用于治疗R/R ALL，缓解率可达70％～90％，但是较高的复发率限制了其临床应用。导致疾病复发的原因之一为含有鼠源单链抗体（scFv）CAR结构的免疫原性[5]–[6]。而人源化CAR-T有望通过降低CAR的免疫原性，延长CAR-T细胞体内存活时间，从而提高CAR-T疗效[7]–[11]。2016年起我中心开展靶向CD19人源化CAR-T细胞（hCART19s）治疗R/R ALL患者的临床试验（NCT02782351），在全年龄段患者中已观察到较好疗效及安全性[7]–[8],[11]。由于在化疗时代，儿童及青少年与成人ALL的治疗与预后存在较大差异。本研究我们回顾性筛选入组临床实验病例中25岁以下的儿童及青少年患者，探讨靶向CD19人源化CAR-T细胞（hCART19s）治疗儿童及青少年（25岁以下）R/R ALL的疗效及安全性。

## 病例与方法

1. 病例资料：筛选2016年5月至2021年9月入组NCT02782351临床试验中31例25岁以下儿童及青少年R/R ALL患者的临床资料。排除标准：①100 d内接受过异基因造血干细胞移植；②合并重要脏器功能障碍、现存严重过敏或既往过敏史（尤其是对IL-2过敏）；③患有传染性疾病。本研究方案通过徐州医科大学附属医院伦理委员会的批准（伦理批号：XYFY2016-KL002-01）。

2. hCART19s细胞的制备和输注：人源化抗CD19单链抗体（scFv）可变区、CD8跨膜区域、CD8铰链区、4-1BB共刺激域、CD3ζ 膜内信号区以及T2A-EGFRt 序列组合成CAR细胞结构，构建慢病毒载体，包装病毒后感染患者来源的CD3^+^ T细胞，获得hCART19s细胞[12]。回输hCART19s细胞前5天采用FC方案进行淋巴细胞清除性预处理，具体为：环磷酰胺750 mg/m^2^，−5 d；氟达拉滨30 mg·m^−2^·d^−1^，−5～−2 d。CAR-T细胞回输当天设为第0天，回输量为1×10^6^个细胞/kg。输注CAR-T细胞前予非激素类抗过敏药物预防过敏反应。

3. 疗效及不良反应评估：完全缓解（CR）、完全缓解兼部分血细胞计数缓解（CRi）、未缓解（NR）均通过NCCN 2016版ALL治疗指南标准进行定义和评估。在hCART19s输注前后定期检测患者外周血IL-2、IL-6、IFN-γ和TNF-α浓度。CAR DNA拷贝数通过实时聚合酶链反应技术（qPCR）检测进行评估。微小残留病（MRD）定义为流式细胞仪中骨髓原始细胞<0.01％或qPCR检测中融合基因的消失。

CAR-T治疗后最常见的不良反应为细胞因子释放综合征（CRS）和神经毒性，CRS分级采用Lee诊断分级标准[13]，神经毒性分级采用不良反应事件评价标准（CTCAE）4.03版本。伴随有髓外疾病的患者，其髓外病灶的检测及评估主要通过体格检查和影像学检测完成。

4. 统计学处理：采用SPSS 22.0软件进行统计学分析。总生存（OS）期定义为CAR-T细胞数输注到患者死亡或末次随访的时间。无事件生存（EFS）期定义为疾病诊断到患者疾病进展、复发、死亡或末次随访时间。OS和EFS主要通过Kaplan-Meier方法绘制生存曲线，Log-rank检验比较组间差异。*P*<0.05为差异有统计学意义。

## 结果

1. 患者特征：31例儿童及青少年R/R ALL患者中，男19例，女12例，中位年龄13（3～24）岁。hCART19s治疗前患者既往治疗线数为2（2～6）次。17例（54.8％）患者既往经历过原发疾病复发，其中1例在异基因造血干细胞移植后复发；4例曾在我中心接受过鼠源性CD19 CAR-T细胞治疗，获得短暂缓解后复发。14例（45.2％）患者表现为难治，其中4例使用标准化诱导缓解方案未达形态学缓解，10例在首次缓解后6个月内发生疾病早期复发。3例患者通过常规染色体核型分析和（或）荧光原位杂交（FISH）检测出Ph染色体的存在，诊断为Ph^+^ ALL，其中1例携带T315I ABL激酶突变。在hCART19s细胞输注前，根据骨髓中原始细胞比例评估患者疾病负荷，20例患者评价为高疾病负荷，即原始细胞比例>20％（18例）或存在髓外侵犯（2例）；11例患者原始细胞比例≤20％，评价为低疾病负荷。患者主要脏器功能均良好，全部患者ECOG评分≤2分。

2. 短期疗效评估：hCART19s细胞治疗后1个月对患者进行短期疗效评估，27例（87.1％）获得CR/CRi，4例（12.9％）为NR。既往接受过鼠源性CAR-T治疗的4例患者中，1例CR，3例NR。在可评估的27例CR/CRi患者中，25例MRD阴性（MRD^−^），2例MRD阳性（MRD^+^）。

3. 不良反应：CRS发生率为77.4％（24/31），中位发生时间为输注后7（0～14）d，中位持续时间为9（1～16）d。轻度CRS（1～2级）20例（64.5％），主要表现为发热、乏力、心动过速等；严重CRS（3～4级）4例（12.9％），以低血压、低氧血症、脏器毒性为主要临床特征；无患者出现5级CRS。所有CRS患者均根据指南接受了密切临床监护和对症支持治疗，4例单独应用托珠单抗治疗，8例单独接受糖皮质激素治疗，12例应用托珠单抗+糖皮质激素联合治疗。治疗后CRS相关症状均缓解，无患者需要ICU监护，无患者因CRS死亡。hCART19s细胞输注后8周内，2例患者发生神经毒性，均表现为轻度可逆性（1级）神经毒性，经对症支持治疗后症状均逐渐消失。

治疗过程中定期检测了24例CRS患者血浆IL-6及C反应蛋白（CRP）水平。1～2级CRS患者（20例）IL-6和CRP峰值中位数分别为15.4（2.7～491.5）ng/L和15.3（0.8～152.0）mg/L，3～4级CRS患者（4例）IL-6和CRP峰值中位数分别为5 000（959～5 000）ng/L和122.3（18.9～200.0）mg/L。3～4级CRS患者的血清IL-6和CRP峰值水平均明显高于1～2级CRS患者（*P*<0.001，*P*＝0.014）。

4. 长期生存状态：中位随访19.3（2.2～62.4）个月，31例儿童及青少年患者的中位EFS时间为15.7（95％ *CI* 8.9～22.5）个月，中位OS时间为32.2（95％ *CI* 10.6～53.9）个月。输注hCART19s后，25例MRD^−^ CR/CRi患者的中位EFS时间和中位OS时间分别为16.2（95％ *CI* 3.3～29.1）个月和32.2（95％ *CI* 5.5～59.0）个月；而2例MRD^+^ CR/CRi患者在输注后很快发生原发疾病复发，EFS时间分别为4.4个月和1.7个月，OS时间分别为8.1个月和2.8个月。

27例CR/CRi患者中，10例未接受进一步治疗，其中2例仍处于长期无复发生存状态，8例出现疾病复发。在8例复发病例中7例表现为CD19阳性（CD19^+^）复发，其中2例为MRD^+^病例；1例表现为CD19阴性（CD19^−^）复发。17例（65.3％）患者hCART19s治疗后接受了其他治疗，其中4例接受化疗，12例桥接移植，1例接受二次hCART19s治疗。在接受化疗的4例患者中，3例为CD19^+^复发后的挽救性治疗，均短期死亡；1例为巩固性治疗，目前仍处于长期无病生存状态。1例CD19^+^复发患者接受二次hCART19s治疗，诊断为NR后短期死亡。12例患者在CR/CRi期间桥接HSCT作为巩固性治疗，从hCART19s输注到桥接移植的中位时间为2.3（1.0～5.9）个月。截至随访终点，移植后8例患者持续处于无复发生存状态，2例CD19^+^复发，2例分别死于移植物抗宿主病（GVHD）和弥漫性血管内凝血（DIC）。长期随访结果显示桥接移植患者EFS和OS均优于未桥接移植患者［EFS：（75.0±12.5）％对（21.1±9.4）％，*P*＝0.010；OS：（75.0±12.5）％对（24.6±10.2）％，*P*＝0.012］。分析既往治疗线数对生存的影响，结果显示既往治疗线数>3次的患者，hCART19s治疗后EFS和OS率明显低于治疗线数≤3次的患者［EFS：0对（49.5±10.4）％，*P*<0.001；OS：0对（52.0±10.8）％，*P*<0.001］。

## 讨论

目前，多项鼠源性CD19 CAR-T疗法治疗R/R ALL的临床试验已证实其显著临床疗效。美国纪念斯隆凯特琳癌症中心进行的一项Ⅰ期临床试验中，53例成人ALL患者接受了19-28z CAR-T细胞治疗，输注后28 d CR率可达83％。中位随访29个月，中位EFS时间为6.1个月，中位OS时间为12.9个月[14]。ELIANA Ⅱ期临床试验中招募了92例患者R/R ALL的儿童和青少年患者，最终75例患者接受了tisagenlecleucel的输注，3个月的疾病总缓解率为81％，中位随访时间分别为13.3个月和12个月，1年OS率和EFS率分别为76％和50％[4]。本研究中，31例儿童及青少年R/R ALL患者接受hCART19s治疗后获得较高CR率（87.1％）。中位随访19.3个月，中位EFS和OS时间分别为15.7个月和32.2个月，研究结果显示了hCART19s治疗儿童及青少年R/R ALL患者具有较好的疗效。

CAR-T细胞中非人源化结构诱发宿主产生免疫反应的能力称为CAR-T细胞的免疫原性，CAR-T细胞潜在免疫原性可能导致CAR-T细胞体内扩增能力下降及存活时间缩短，诱导原发疾病复发从而影响疗效及安全性[15]–[19]。人源化修饰是将鼠源性scFv结构区保留，仅将骨架区改为人源化序列，这在减少机体对CAR-T产生免疫反应的同时保留了与鼠源性抗体相似的对靶向抗原的亲和力。人源化修饰CAR结构可有效降低CAR-T细胞的免疫原性，延长CAR-T体内存活时间，提高CAR-T免疫疗法的有效性[11],[20]–[22]。尽管在本次临床试验中包括CR率、EFS率和OS率在内的临床疗效似乎与既往鼠源性CAR-T的临床试验的结果相当，但是本临床试验未进行随机对照，很难明确鼠源性或人源化CD19 CAR-T哪种疗效更好。

Park等[14]报道认为治疗前疾病负荷、MRD状态和既往治疗线数可影响CAR-T治疗长期生存率和反应的持久性。在本试验中，生存分析结果显示桥接移植对EFS和OS产生积极影响，而既往治疗线数对长期生存产生负面影响。由于本临床试验存在样本量少、随访时间短等局限性，未来还需要扩大样本量、延长随访时间来进一步证实相关因素对hCART19s疗效的影响。

CRS和神经毒性是CAR-T治疗后最常见的不良反应。既往多项应用CD19 CAR-T细胞疗法治疗R/R ALL的临床试验报道了CRS的发生率（56％～100％）以及严重CRS（3～5级）的发生率（3％～71％）较高，但是不同临床试验中神经毒性发生率具有较大差异（29％～72％）[4],[7],[23]–[28]。本项研究中，24例（77.4％）患者出现CRS，大部分患者表现为1～2级CRS，仅4例患者出现3～4级CRS，所有患者经有效治疗后相关症状均得到缓解。值得注意的是，本试验中仅2例患者出现1级神经毒性，该结果显著低于既往研究[29]–[30]。考虑可能与本组病例输注的CAR-T细胞的剂量较低以及对不良反应的早期干预（如托珠单抗等）有关。

总之，本研究结果显示，hCART19s治疗儿童及青少年R/R ALL患者安全有效，严重不良反应的发生率低且可控，hCART19s细胞输注后桥接移植患者长期疗效更优。
